# Longitudinal association between personality traits and homebound status in older adults: results from the National Health and Aging Trends Study

**DOI:** 10.1186/s12877-022-02771-8

**Published:** 2022-02-02

**Authors:** Xiaocao Sun, Siyuan Tang, Christina E. Miyawaki, Yuxiao Li, Tianxue Hou, Minhui Liu

**Affiliations:** 1 Xiangya School of Nursing, Central South University, 172 Tongzipo Road of Yuelu District, Changsha, 410013 Hunan China; 2 Graduate College of Social Work, University of Houston, Houston, TX USA

**Keywords:** Personality traits, Homebound, Older adults, Conscientiousness

## Abstract

**Background:**

Personality is associated with predictors of homebound status like frailty, incident falls, mobility, and depression. However, the relationship between personality traits and homebound status is unclear. This study aims to examine the longitudinal association between personality traits and homebound status among older adults.

**Methods:**

Using data of non-homebound community-dwelling adults aged 65 years and older in the 2013 and 2014 waves (baseline) of the National Health and Aging Trends Study (*N* = 1538), this study examined the association between personality traits and homebound status. Homebound status (non-homebound, semi-homebound, and homebound) was determined by the frequency of going outside, difficulty in going outside, and whether there was help when going outside. Personality traits, including conscientiousness, extraversion, neuroticism, openness, and agreeableness were assessed using the 10-item Midlife Development Inventory on a rating scale from 1 (not at all) to 4 (a lot). Ordered logistic regression models were used to examine whether personality traits predicted homebound status in later 3 years with and without adjusting covariates.

**Results:**

The sample was on average 77.0 ± 6.70 years old, and 55% were female. The majority were non-Hispanic whites (76%), and received some college or vocational school education or higher (55%). Homebound participants tended to be less educated older females. Three years later, 42 of 1538 baseline-non-homebound participants (3%) became homebound, and 195 participants (13%) became semi-homebound. Among these five personality traits, high conscientiousness (adjusted odds ratio [OR] = 0.73, *p* < 0.01) was associated with a low likelihood of becoming homebound after adjusting demographic and health-related covariates.

**Conclusions:**

These findings provided a basis for personality assessment to identify and prevent individuals from becoming homebound.

**Supplementary Information:**

The online version contains supplementary material available at 10.1186/s12877-022-02771-8.

## Background

Homebound status is a condition in which daily activities are confined at home [1]. Approximately 2 million older adults in the United States are homebound, including about 400,000 people who are completely homebound and 1.6 million people who rarely go out [2]. Homebound status is associated with numerous adverse health outcomes, such as functional impairments [3], multimorbidity [2], frailty [4], falls [5], and depression [6]. Homebound individuals were also more likely to experience hospitalization [2]. These negative health outcomes tend to burden both homebound individuals and their caregivers, as well as lead to substantial health care costs [7], presenting major challenges in health care systems. Established risk factors for homebound status include advanced age, female gender, low education level [8], poor physical health, psychological stress and less social support [9, 10]. Personality – an enduring set of traits and characteristics that influence one’s thoughts, feelings, and behavior [11] – may also influence the homebound status.

There are five major personality traits known as the Five Factor Model (FFM): conscientiousness (the tendency to be organized, responsible, industrious and disciplined), extraversion (the tendency to be sociable, outgoing and energetic), neuroticism (the tendency to experience negative emotions), openness (the tendency to be curious, creative, open to new ideas and intellectual), and agreeableness (the tendency to be kind, warm, tolerant and affable) [12]. Previous studies have reported that these personality traits have been shown to predict multiple health risk factors that are related to homebound status [13, 14]. In terms of physical health, personality traits were associated with functional ability [15], cognitive decline, chronic diseases [16], and pain [17, 18]. For example, evidence suggests that older adults with low conscientiousness and high neuroticism tended to engage in fewer physical activities [19], have poorer physical function [20], higher risk of falling [21] and frailty [22]. Individuals with low extraversion were found to be associated with poor mobility performance [23]. Those with low openness were shown to be associated with limited physiological reserve leading to the onset or progression of frailty [24]. In terms of mental health, personality traits were associated with depression and anxiety. For instance, older adults with high neuroticism were more likely to experience negative emotions and present depressive symptoms [16, 18, 25, 26]. Lower extraversion and conscientiousness were significantly associated with the presence and severity of depression [27]. Neuroticism was positively, and extraversion, conscientiousness were inversely, associated with anxiety disorder [28]. In addition, certain personality traits also relate to health behaviors which might lead to homebound status. Individuals with higher extraversion, higher neuroticism, lower conscientiousness and lower agreeableness were more likely to engage in risky health behaviors like smoking [29–31], which has been known to increase the risk of frailty and eventual disability [32]. Taken all together, certain personality traits may share related risk factors of homebound status (including physical, mental, and health-related behavioral factors).

Despite these established associations between personality traits and risk factors of homebound status, it is still unclear what specific personality traits among conscientiousness, extraversion, neuroticism, openness and agreeableness are associated with older adults becoming homebound. It is important to understand this association because first, it will provide a scientific basis for effectively identifying individuals at risk of becoming homebound by using relevant tools (e.g., a personality assessment). Second, it will help health care professionals to establish relevant preventive strategies for homebound older adults (e.g., an educational intervention program). Third, it will guide the targeted interventions aiming at reducing homebound risks and improving the quality of life of older adults. Therefore, this study aimed to describe the characteristics of older adults with different homebound status (non-homebound, semi-homebound and homebound) and determine what specific personality traits are associated with becoming homebound.

## Methods

### Design and sample

Data were drawn from the National Health and Aging Trends Study (NHATS), a longitudinal cohort study of Medicare beneficiaries in the United States aged 65 and older. We combined the cross-sectional data of 1538 non-homebound community-dwelling older adults in Round 3 (R3, Year 2013) and Round 4 (R4, Year 2014) who provided complete data on personality traits and homebound status as baseline information. Data on homebound status in R6 (three-year follow up for R3 participants) and homebound status data in R7 (three-year follow up for R4 participants) were used for follow-up prediction. To examine the longitudinal associations between personality traits and homebound status, we only included non-homebound participants in R3 and R4, and used personality traits data in R3 and R4 to predict the incidence of homebound in R6 and R7, respectively. The Johns Hopkins University Institutional Review Board approved the study protocol and written informed consent was obtained from all participants or their proxy respondents in the NHATS study.

### Measures

#### Dependent variable

The dependent variable of this study is homebound status. There have been some definitions of homebound status from perspectives of different stakeholders (e.g., Medicare center, researchers, community-based health service providers) [3]. Since no gold standard measurement had been built yet and NHATS has no pre-defined measure of homebound status, we used the measure of homebound status developed by Ornstein and colleagues [2], which had several advantages. It was grounded on gerontological conceptual frameworks in which both personal capacity and social support were considered related to disability [33, 34]. It also helped to more accurately estimate the homebound population in the U.S. and to develop targeted programs that serve the homebound individuals [2]. Last but not least, it was exactly applicable for our study.

**Fig. 1:**
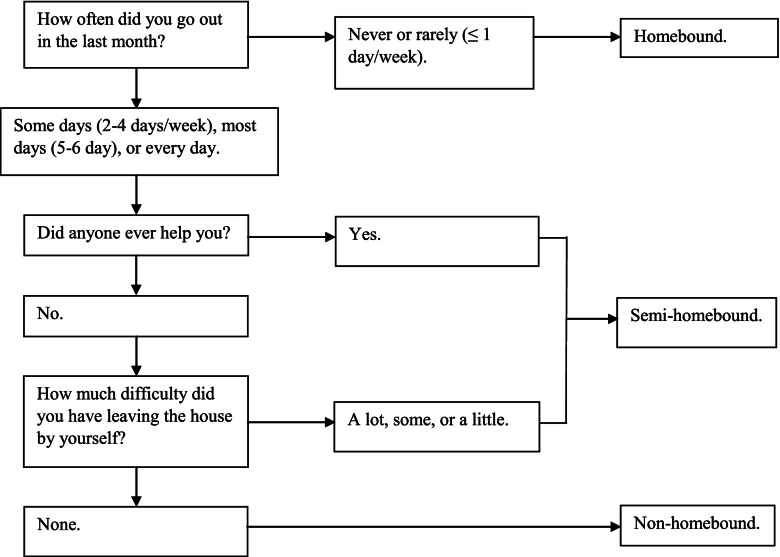
Determining Homebound Status Using the National Health and Aging Trends Study

We classified individuals into three homebound status categories: (1) non-homebound; (2) semi-homebound; and (3) homebound. Responses from three questions were used in NHATS to determine the classification. First, the participants were asked, “How often did you go out in the last month?” Answers included every day, most days (5–6 days a week), some days (2–4 days a week), rarely (once a week or less), and never. Persons who went outside were then inquired whether anyone helped with going outside. Those without other’s help were also asked to report difficulty level (none, a little, some, and a lot) of leaving the house by themselves (Fig. 1).

Participants were considered non-homebound if they went out at least twice per week without others’ help and without difficulty. If participants went out with the same frequency as non-homebound counterparts, but they needed others’ help or had difficulty leaving the house by themselves, semi-homebound was categorized. Homebound was classified if they rarely (once a week or less) or never went out in the last month.

#### Independent variables

Five major personality traits - conscientiousness, extraversion, neuroticism, openness, and agreeableness - were assessed in 2013 for one-third of the sample and in 2014 for the second third using the 10-item Midlife Development Inventory [35, 36], which has been used in a previous study showing an association between personality and motoric cognitive risk syndrome [37]. Respondents were asked the degree to which each adjective described them, rating each one on a four-point Likert scale (1 = not at all; 2 = a little; 3 = some; 4 = a lot). The ten adjectives describing each personality trait were: “organized” and “thorough” for conscientiousness, “outgoing” and “talkative” for extraversion, “worrying” and “nervous” for neuroticism, “creative” and “imaginative” for openness, and “warm” and “caring” for agreeableness. Each score was calculated by the average of the ratings.

#### Covariates

All potential confounders that might influence the association between personality traits and homebound status were identified as covariates based on literature review. Demographic and health-related covariates were listed as follows.

##### Demographic characteristics

These included age, gender, race/ethnicity (non-Hispanic white, non-Hispanic Black, Hispanic, and other), education (less than high school, high school graduates, some college or vocational school, bachelor or higher degrees), and living arrangement (alone, with spouse only, with others only, with spouse and others).

##### Health-related factors

1) Numbers of activities of daily living (ADL) difficulties were counted in eating, dressing, bathing, and toileting. 2) Numbers of chronic illnesses were determined by whether the person has ever been told by a doctor that he/she has any conditions such as heart attack, heart disease, high blood pressure, arthritis, osteoporosis, diabetes, lung disease, stroke, and cancer. 3) Dementia was determined by asking participants whether they have ever been told by a doctor that he/she has dementia or Alzheimer’s disease. 4) Anxiety and 5) Depression were assessed by the Generalized Anxiety Disorder-2 (GAD-2) scale and the Patient Health Questionnaire-2 (PHQ-2) scale, respectively. Cronbach’s alpha for the GAD-2 and the PHQ-2 were 0.71 and 0.77, respectively [38, 39]. The two scales have been validated in elderly people [38, 40]. A cut-off point of 3 and higher was used to determine anxiety and depressive symptoms [41, 42]. 6) Pain was determined by asking whether participants were bothered by pain in the last month. 7) Hospitalization was assessed by asking whether they had overnight hospital stays within the last 12 months.

### Statistical analysis

We used mean and standard deviation (SD) to describe continuous variables including age and the scores of five personality traits among older adults. Frequencies and proportions were used to describe categorical variables, such as demographic characteristics and health-related factors. Chi-square test, one-way analysis of variance (ANOVA) and Bonferroni’s correction were used to compare demographic characteristics and health-related factors, and the scores of five personality traits in all participants with different homebound status. We also examined the correlations among all study variables using pairwise correlations.

To examine the longitudinal association between personality traits and homebound status, we used ordered logistic regression models with homebound status in later three waves treated as the outcome and personality traits as the main predictors. For each personality trait, we first analyzed the crude association without any adjustment of covariates (Model 1), then adjusted for demographic characteristics (Model 2) and finally, further adjusted for health-related factors (Model 3). The proportional odds assumption was tested in each model using the “gologit2” command [43], and all models met this assumption. To increase the robustness of our findings, we conduct four types of sensitivity analyses in addition to the primary analysis. First, instead of examining each trait separately, we included all five traits simultaneously as independent variables in crude and adjusted models. Second, we also examined the cross-sectional association using combined data of participants in R3 and R4 as the sample size is larger and it increased the power (*N* = 2788; homebound, semi-homebound and non-homebound sample were 279, 494, and 2075, respectively). Third, longitudinal analysis with longer follow-up period were also conducted using personality traits data in R3 and R4 to predict the incidence of becoming homebound in R9 and R10 (the newest round data of NHATS). A total of 1144 older adults were included (869 non-homebound, 197 semi-homebound, and 78 homebound older adults). Lastly, we used multinominal logistic regression to examine the pairwise differences among three different homebound status groups. Odds ratios (OR), 95% confidence intervals (CI), and relative risk ratio (RRR) were reported. For all the regressions and sensitivity analyses we did, we calculated an effect size using Cohen’s ƒ^2^, which is equal to R^2^/(1-R^2^). By convention, ƒ^2^ effect sizes of 0.02, 0.15, and 0.35 are termed small, medium, and large, respectively [44]. Given that missing values on covariates were less than 3%, we did not apply any techniques to handle missing data. All tests were two-tailed and statistical significance was defined as a value of *p* < 0.05. All statistical analyses were performed using Stata version 15.0.

## Results

### Sample characteristics

Table 1 shows baseline demographics, health-related covariates, and personality traits of the participants grouped by three-year-later homebound status. The sample was on average 77.0 ± 6.70 years old, and 55% were female. The majority were non-Hispanic whites (76%), followed by non-Hispanic Blacks (17%). More than half of the sample (55%) received some college or vocational school education or higher, and 32% lived alone. The vast majority of the sample (91%) reported having at least one chronic illness and many (82%) had been hospitalized in the last 12 months, but not experiencing anxiety (94%) and depression (94%). Three years later, 42 of 1538 baseline-non-homebound participants (3%) became homebound, and 195 participants (13%) became semi-homebound.

**Table 1:** Baseline characteristics of participants by follow-up homebound status

Characteristics	Total (*N* = 1538)	Homebound (*n* = 42, G1)	Semi-homebound (*n* = 195, G2)	Non-homebound (*n* = 1301, G3)	F or χ^2^	*p* values	*p* values for paired comparisons		
							G1 vs G2	G1 vs G3	G2 vs G3
Age, mean (SD†), years	77.0 (6.70)	82.1 (6.59)	80.1 (7.26)	76.4 (6.41)	40.43	< 0.001	0.217	< 0.001	< 0.001
Gender, n (%)					13.47	0.001	0.032	0.001	0.049
Female	848 (55.1)	33 (78.6)	119 (61.0)	696 (53.5)					
Male	690 (44.9)	9 (21.4)	76 (39.0)	605 (46.5)					
Race, n (%)					20.64	0.002	0.209	0.003	0.037
White, non-Hispanic	1174 (76.3)	23 (54.8)	139 (71.3)	1012 (77.8)					
Black, non-Hispanic	263 (17.1)	15 (35.7)	46 (23.6)	202 (15.5)					
Hispanic	63 (4.1)	3 (7.1)	7 (3.6)	53 (4.1)					
Other	38 (2.5)	1 (2.4)	3 (1.5)	34 (2.6)					
Education, n (%)					25.22	< 0.001	0.573	0.005	0.002
Less than high school	295 (19.3)	16 (38.1)	54 (27.7)	225 (17.4)					
High school graduates	402 (26.3)	11 (26.2)	54 (27.7)	337 (26.0)					
Some college or vocational school	387 (25.3)	8 (19.0)	43 (22.1)	336 (26.0)					
Bachelor or higher	447 (29.2)	7 (16.7)	44 (22.6)	396 (30.6)					
Living arrangement, n (%)					24.86	< 0.001	0.177	0.003	0.006
Alone	500 (32.5)	17 (40.5)	78 (40.0)	405 (31.3)					
With spouse/partner only	690 (44.9)	10 (23.8)	66 (33.8)	614 (47.2)					
With others only	208 (13.5)	12 (28.6)	30 (15.4)	166 (12.8)					
With spouse/partner and with others	140 (9.1)	3 (7.1)	21 (10.8)	116 (8.9)					
Number of ADL† difficulties, mean (SD†)	0.17 (0.47)	0.33 (0.61)	0.35 (0.65)	0.14 (0.42)	86.87	< 0.001	1.000	0.022	< 0.001
Number of chronic illnesses, n (%)					29.90	< 0.001	0.882	0.058	< 0.001
0	138 (9.0)	1 (2.4)	7 (3.6)	130 (10.0)					
1–3	1137 (73.9)	30 (71.4)	133 (68.2)	974 (74.9)					
4+	263 (17.1)	11 (26,2)	55 (28.2)	197 (15.1)					
Dementia, n (%)					19.89	< 0.001	0.190	< 0.001	0.002
Yes	47 (3.1)	5 (11.9)	12 (6.2)	30 (2.3)					
No	1491 (96.9)	37 (88.1)	183 (93.9)	1271 (97.9)					
Anxiety symptom, n (%)					13.01	0.001	0.382	0.577	< 0.001
Yes	93 (6.1)	3 (7.1)	23 (11.8)	67 (5.2)					
No	1435 (93.9)	39 (92.9)	172 (88.2)	1224 (94.8)					
Depressive symptom, n (%)					4.48	0.106			
Yes	98 (6.4)	37 (92.5)	175 (90.2)	1224 (94.2)					
No	1436 (93.6)	3 (7.5)	19 (9.8)	76 (5.9)					
Pain, n (%)					4.70	0.095			
Yes	771 (50.1)	16 (38.1)	88 (45.1)	663 (51.0)					
No	767 (49.9)	26 (61.9)	107 (54.9)	638 (49.0)					
Hospitalization, n (%)					17.94	< 0.001	0.570	0.011	< 0.001
Yes	282 (18.4)	13 (31.7)	53 (27.3)	216 (16.6)					
No	1254 (81.6)	28 (68.3)	141 (72.7)	1085 (83.4)					
Personality traits, mean (SD†)									
Conscientiousness	3.32 (0.65)	3.21 (0.85)	3.14 (0.71)	3.35 (0.63)	9.18	< 0.001	1.000	0.565	< 0.001
Extraversion	3.21 (0.72)	3.14 (0.78)	3.11 (0.75)	3.23 (0.71)	2.62	0.073			
Neuroticism	2.15 (0.82)	2.10 (0.86)	2.13 (0.87)	2.15 (0.81)	0.14	0.869			
Openness	2.92 (0.81)	2.67 (0.90)	2.84 (0.86)	2.94 (0.80)	3.29	0.038	0.632	0.101	0.353
Agreeableness	3.60 (0.51)	3.49 (0.66)	3.59 (0.59)	3.60 (0.49)	0.98	0.377			

^†^*SD* Standard Deviation, *ADL* Activities of Daily Living

Except for depressive symptoms and pain, all demographic characteristics and health-related factors significantly differed by homebound status. The homebound older adults compared to non-homebound counterparts were older (82.1 vs 76.4 years old), more likely to be female (78.6% vs 53.5%), have dementia (11.9% vs 2.3%), reported more ADL difficulties (mean of 0.33 vs 0.14) and experienced anxiety (7.1% vs 5.2%). Homebound older adults compared to semi-homebound and non-homebound older adults also tended to be less-educated (38.1% vs 27.7% vs 17.4%) and had more chronic illnesses (97.6% vs 96.4% vs 90.0%).

The average scores of conscientiousness, extraversion, neuroticism, openness, and agreeableness were 3.32 ± 0.65, 3.21 ± 0.72, 2.15 ± 0.82, 2.92 ± 0.81, and 3.60 ± 0.51, respectively. The difference of conscientiousness (3.21 ± 0.85 vs 3.14 ± 0.71 vs 3.35 ± 0.63, *p* < 0.001) and openness (2.67 ± 0.90 vs 2.84 ± 0.86 vs 2.94 ± 0.80, *p* = 0.038) among the three homebound status was statistically significant. The other three personality traits of extraversion, neuroticism, and agreeableness did not differ among different homebound status groups.

### Longitudinal association between personality traits and homebound status

The summary of the ordinal logistic regressions of personality traits predicting homebound status is presented in Table 2. Conscientiousness was significantly associated with homebound status in Model 1 (OR, 95% CI = 0.65, 0.54–0.80), Model 2 (OR, 95% CI = 0.71, 0.57–0.87) and Model 3 (OR, 95% CI = 0.73, 0.59–0.91) meaning that older adults with higher conscientiousness were less likely to become homebound. The association between extraversion and homebound status was statistically significant in Model 1 (OR, 95% CI = 0.81, 0.67–0.97), but became insignificant after adjusting for demographic covariates in Model 2 (OR, 95% CI = 0.84, 0.69–1.02) and further adjusting for health-related factors in Model 3 (OR, 95% CI = 0.88, 0.72–1.07). Neuroticism was not significantly associated with homebound status in Model 1 (OR, 95% CI = 0.96, 0.81–1.14) and Model 2 (OR, 95% CI = 0.94, 0.79–1.10), but showed a significant association in Model 3 (OR, 95% CI = 0.81, 0.67–0.98). Openness was significantly associated with homebound status in Model 1 (OR, 95% CI = 0.82, 0.69–0.97), but not in Model 2 (OR, 95% CI = 0.92, 0.77–1.10) and Model 3 (OR, 95% CI = 0.88, 0.74–1.06). Agreeableness was the only personality trait that did not show any statistically significant association with homebound status in all three models: Model 1 (OR, 95% CI = 0.90, 0.69–1,17), Model 2 (OR, 95% CI = 0.84, 0.64–1.11), and Model 3 (OR, 95% CI = 0.85, 0.63–1.12). The effect sizes were about 0.01 for Model 1, ranged from 0.08 to 0.09 for Model 2, and ranged from 0.12 to 0.13 for Model 3. Overall, the effect sizes were small or medium for prediction from each personality trait to three-year-later homebound status. A matrix of correlation coefficients of independent variables was presented in Supplemental Table 1.

**Table 2:** Summary of ordinal logistic regression of personality traits predicting homebound status (3 years follow up)

Variables	Model 1 OR (95% CI)†	Effect size (*f*^2^)	Model 2 OR (95% CI)†	Effect size (*f*^2^)	Model 3 OR (95% CI)†	Effect size (*f*^2^)
Conscientiousness	0.65 (0.54, 0.80) ***	0.01	0.71 (0.57, 0.87) ***	0.09	0.73 (0.59, 0.91) **	0.13
Extraversion	0.81 (0.67, 0.97) *	0.01	0.84 (0.69, 1.02)	0.08	0.88 (0.72, 1.07)	0.12
Neuroticism	0.96 (0.81, 1.14)	0.01	0.94 (0.79, 1.12)	0.08	0.81 (0.67, 0.98) *	0.13
Openness	0.82 (0.69, 0.97) *	0.01	0.92 (0.77, 1.10)	0.08	0.88 (0.74, 1.06)	0.12
Agreeableness	0.90 (0.69, 1.17)	0.01	0.84 (0.64, 1,11)	0.08	0.85 (0.63, 1.12)	0.12

Note: Model 1: crude association

Model 2: Model 1 + demographic covariates (age, gender, race, education, living arrangement)

Model 3: Model 1 + demographic and health-related covariates (ADL difficulties, number of chronic illnesses, dementia, depression, anxiety, pain, and hospitalization)

^**†**^*OR* Odds ratio, *CI* confidence interval; *p < 0.05; **p < 0.01; ***p < 0.001

### Sensitivity analyses of personality traits predicting homebound status

Table 3 presents the results of analysis with all traits included simultaneously, which were similar to those with each trait separately. Conscientiousness was the only trait significantly associated with homebound status in three models: Model 1 (OR, 95% CI = 0.68, 0.55–0.84), Model 2 (OR, 95% CI = 0.72, 0.57–0.90), and Model 3 (OR, 95% CI = 0.76, 0.60–0.95). Neuroticism was only significantly associated with homebound status in Model 3 (OR, 95% CI = 0.81, 0.67–0.99). Personality traits of extraversion, openness and agreeableness were not associated with homebound status in any models.

**Table 3:** Summary of ordinal logistic regression of personality traits predicting homebound status (3 years follow up)

Variables	Model 1 OR (95% CI)†	Effect size (*f*^2^)	Model 2 OR (95% CI)†	Effect size (*f*^2^)	Model 3 OR (95% CI)†	Effect size (*f*^2^)
Conscientiousness	0.68 (0.55, 0.84) ***	0.01	0.72 (0.57, 0.90) **	0.09	0.76 (0.60, 0.95) *	0.13
Extraversion	0.87 (0.70, 1.07)		0.89 (0.71, 1.11)		0.94 (0.75, 1.17)	
Neuroticism	0.95 (0.80, 1.12)		0.93 (0.78, 1.12)		0.81(0.67, 0.99) *	
Openness	0.92 (0.76, 1.11)		1.04 (0.71, 0.85)		0.97 (0.79, 1.19)	
Agreeableness	1.13 (0.84, 1.52)		0.99 (0.73, 1.35)		0.97 (0.71, 1.33)	

Note: All personality variables were entered simultaneously

Model 1: crude association

Model 2: Model 1 + demographic covariates (age, gender, race, education, living arrangement)

Model 3: Model 1 + demographic and health-related covariates (Body Mass Index, self-reported health, number of chronic illnesses, dementia, depression, anxiety, pain, and hospitalization)

*†OR* Odds ratio, *CI* confidence interval; * *p* < 0.05.** *p* < 0.01.*** *p* < 0.001

Table 4 shows the cross-sectional association between personality traits and homebound status using data in R3 and R4. Table 5 presents the longitudinal association using personality traits to predict homebound status in 6 years (results combined from R3 to R9 and R4 to R10). Conscientiousness was still the only trait associated with homebound status in all models in Tables 4 and 5. The differences were as follows. In cross-sectional analysis, the other four traits were associated with homebound status in Model 1 and Model 2, but not in Model 3. In longitudinal analysis with six-year follow up, except for openness could predict homebound status only in crude model, no more significant associations between personality traits and homebound status were found in other models and other traits. Overall, the results of cross-sectional and six-year longitudinal analyses were similar to three-year longitudinal associations.

**Table 4:** Cross-sectional analysis of association between personality traits and homebound status

Variables	Model 1 OR (95% CI)†	Effect size (*f*^2^)	Model 2 OR (95% CI)†	Effect size (*f*^2^)	Model 3 OR (95% CI)†	Effect size (*f*^2^)
Conscientiousness	0.50 (0.45, 0.56) ***	0.04	0.57 (0.50, 0.64) ***	0.17	0.74 (0.64, 0.85) ***	0.33
Extraversion	0.65 (0.58, 0.73) ***	0.01	0.70 (0.62, 0.79) ***	0.15	0.88 (0.76, 1.01)	0.32
Neuroticism	1.46 (1.32, 1.61) ***	0.01	1.46 (1.31, 1.63) ***	0.15	1.07 (0.93, 1.22)	0.32
Openness	0.67 (0.61, 0.75) ***	0.01	0.80 (0.71, 0.90) ***	0.14	0.92 (0.81, 1.04)	0.32
Agreeableness	0.78 (0.67, 0.90) **	0.01	0.73 (0.62, 0.86) ***	0.14	0.92 (0.76, 1.11)	0.32

Note: Model 1: crude association

Model 2: Model 1 + demographic covariates (age, gender, race, education, living arrangement)

Model 3: Model 1 + demographic and health-related covariates (Body Mass Index, self-reported health, number of chronic illnesses, dementia, depression, anxiety, pain, and hospitalization)

*†OR* Odds ratio, *CI* confidence interval; ** *p* < 0.01.*** *p* < 0.001

**Table 5:** Summary of ordinal logistic regression of personality traits predicting homebound status (6 years follow up)

Variables	Model 1 OR (95% CI)†	Effect size (*f*^2^)	Model 2 OR (95% CI)†	Effect size (*f*^2^)	Model 3 OR (95% CI)†	Effect size (*f*^2^)
Conscientiousness	0.74 (0.60, 0.91) **	0.01	0.77 (0.61, 0.95) *	0.06	0.79 (0.63, 0.99) *	0.08
Extraversion	0.86 (0.71, 1.03)	0.01	0.84 (0.69, 1.02)	0.06	0.84 (0.69, 1.02)	0.08
Neuroticism	1.05 (0.89, 1.24)	0.01	1.02 (0.85, 1.22)	0.06	0.99 (0.82, 1.20)	0.08
Openness	0.80 (0.68, 0.94) **	0.01	0.88 (0.73, 1.04)	0.06	0.87 (0.72, 1.04)	0.08
Agreeableness	1.08 (0.81, 1.44)	0.01	0.94 (0.69, 1.27)	0.06	0.94 (0.69, 1.27)	0.08

Note: Model 1: crude association

Model 2: Model 1 + demographic covariates (age, gender, race, education, living arrangement)

Model 3: Model 1 + demographic and health-related covariates (ADL difficulties, number of chronic illnesses, dementia, depression, anxiety, pain, and hospitalization)

^**†**^OR = Odds ratio; CI = confidence interval; *p < 0.05; **p < 0.01

Table 6 shows the pairwise differences among three different homebound status groups. Individuals with higher conscientiousness score had a significantly lower risk of becoming semi-homebound in 3 years in three models (RRRs for Model 1, 2, and 3 were 0.64, 0.68, and 0.70 respectively). Significant baseline extraversion differences were found to be predictive of semi-homebound in crude model (RRR = 0.80), indicating that higher extraversion was associated with lower likelihood of becoming semi-homebound. Higher openness was also associated with lower likelihood of becoming homebound compared with non-homebound older adults in crude model (RRR = 0.67). However, differences of extraversion and openness to predict homebound status became insignificant after controlling for demographics and health-related covariables.

**Table 6:** Summary of multinominal logistic regression of personality traits predicting homebound status

Variables^a^	Model 1 RRR (95% CI)†	Effect size (*f*^2^)	Model 2 RRR (95% CI)†	Effect size (*f*^2^)	Model 3 RRR (95% CI)†	Effect size (*f*^2^)
Conscientiousness		0.01		0.09		0.13
Non-homebound (reference)	1.00		1.00		1.00	
Semi-homebound	0.64 (0.51, 0.79) ***		0.68 (0.54, 0.85) **		0.70 (0.56, 0.89) **	
Homebound	0.74 (0.47, 1.15)		0.82 (0.51, 1.30)		0.80 (0.50, 1.30)	
Extraversion		0.01		0.08		0.12
Non-homebound (reference)	1.00		1.00		1.00	
Semi-homebound	0.80 (0.65, 0.98) *		0.83 (0.67, 1.02)		0.84 (0.68, 1.05)	
Homebound	0.84 (0.56, 1.27)		0.88 (0.57, 1,34)		0.89 (0.57, 1.40)	
Neuroticism		0.01		0.08		0.13
Non-homebound (reference)	1.00		1.00		1.00	
Semi-homebound	0.97 (0.81, 1.17)		0.97 (0.80, 1.18)		0.82 (0.66, 1.01)	
Homebound	0.92 (0.63, 1.34)		0.88 (0.60, 1.29)		0.80 (0.52, 1.23)	
Openness		0.01		0.08		0.12
Non-homebound (reference)	1.00		1.00		1.00	
Semi-homebound	0.86 (0.72, 1.04)		0.96 (0.79, 1.16)		0.92 (0.76, 1.12)	
Homebound	0.67 (0.47, 0.97) *		0.80 (0.54, 1.18)		0.72 (0.49, 1.08)	
Agreeableness		0.01		0.08		0.12
Non-homebound (reference)	1.00		1.00		1.00	
Semi-homebound	0.97 (0.72, 1.31)		0.92 (0.68, 1.25)		0.93 (0.68, 1.27)	
Homebound	0.68 (0.40, 1.17)		0.60 (0.34, 1.04)		0.62 (0.35, 1.10)	

^a^Non-homebound was treated as the base outcome

Model 1: crude association

Model 2: Model 1 + demographic covariates (age, gender, race, education, living arrangement)

Model 3: Model 1 + demographic and health-related covariates (ADL difficulties, number of chronic illnesses, dementia, depression, anxiety, pain, and hospitalization)

*†RRR* Relative Risk Ratio, *CI* confidence interval; *p < 0.05; **p < 0.01; *** *p* < 0.001

Overall, the results of sensitivity analyses were similar to above ordinal logistic regressions, and the effect sizes in the sensitivity analyses were small or medium.

## Discussion

Using data from a large-sample cohort study, we examined the association between five personality traits (conscientiousness, extraversion, neuroticism, openness, and agreeableness) and homebound status (non-homebound, semi-homebound, and homebound) in older adults. To our knowledge, this is the first study to examine how specific personality traits are associated with becoming homebound. We found that the average scores of conscientiousness differed significantly across the three different home-bound status. Furthermore, conscientiousness appeared to independently predict the participants’ three-year-later homebound status. This finding supports the previous studies indicating a possible association between personality traits and risk of becoming homebound due to the participants’ risky physical (e.g., poor mobility [23], frailty [24]), mental conditions (e.g., depression [45]), and risky health-related behaviors (e.g., smoking [29]) [13–32].

Our study found that older adults with high conscientiousness were less likely to be homebound. In fact, it was the strongest protective factor from becoming homebound among the five personality traits in our study. This finding is consistent with the results of a meta-analysis of 16 large national datasets (*N* > 125,000) reporting that older adults with high conscientiousness might perceive fewer barriers to exercising and thus, avoid homebound-related behaviors such as physical inactivity and sedentary lifestyle [19]. Individuals with high conscientiousness were also less likely to be smokers, which might lead to being frail, disable and homebound [29–31]. High conscientiousness was also found to be a protective factor from depression [27, 45], which could increase the risk of becoming homebound [6]. Furthermore, Stephan et al.’s study found that higher conscientiousness was associated with a lower likelihood of the motoric cognitive risk syndrome [37], which was associated with disability implying homebound status [46].

High extraversion and high openness were showed to be protective factors from becoming homebound without adjusting any covariables in our study. However, both associations became insignificant after adjusting for demographic factors (Model 2) and further adjusting for health-related covariates (Model 3). Agreeableness was not associated with homebound status in all three models (Model 1,2,3). Evidence did exist and indicated possible associations between these three personality traits and homebound status. One previous study suggested that high extraversion was associated with better mobility performance [23], preventing from becoming homebound. Another study found a strong association between higher openness and an active lifestyle avoiding becoming homebound [24]. High agreeableness was suggested to be associated with a physically active lifestyle and certain health behaviors, both of which decreased the likelihood of becoming homebound [24, 29–31]. However, these studies used cross-sectional analysis, in which predictive effects of personality traits cannot be obtained. A variety of measurement tools for personality traits were used, and this could affect the generalization of these studies. In addition, complex interactions among personality traits might play a role in forming their associations with homebound status. Therefore, it is safer to conclude that these three personality traits were not associated with homebound status in this study.

Neuroticism was not significantly associated with homebound status in Model 1 and Model 2, but showed a significant association in Model 3. This significant association was unexpected as a large number of previous literatures did not support this finding. First, neuroticism has been shown to be associated with negative health and emotional outcomes in many studies [13, 21, 25], but seemed to be a protective factor from becoming homebound in this study. Second, the OR is very close to 1, which is a sign of an irrelevant factor. High neuroticism did show a link with poor physical function [20] and risk of frailty [22] in previous studies, which may contribute to homebound status. However, these studies failed to clarify the potential mechanism underlying the statistically significant associations, which could help to explain our findings. For example, the association between neuroticism and frailty could be partly contributed by their genetic overlap and biological mechanism through the immune-endocrine system [22], which may not play an equivalent role in the association between neuroticism and homebound status. Therefore, it was possible that neuroticism was not associated with homebound status.

The sensitivity analyses increased the robustness of our findings. Including five personality traits simultaneously as independent variables in regression models supported results of the primary analysis. The cross-sectional analysis with a larger sample size and longitudinal analysis with a longer period follow-up also further supported the primary analysis. Multinominal logistic regression provided new information of pairwise differences among three different homebound status groups.

In multinominal logistic regression analysis, conscientiousness differed significantly between non-homebound and semi-homebound group in all three models. Furthermore, conscientiousness was also the variable that kept the most consistent association with homebound status across models. This finding underscores the importance of screening in older adults early and identify individuals who are at a higher risk of being homebound as early as we can. It was possible that conscientiousness played a more important role at an early stage of becoming homebound rather than a later stage. We also found significant differences in crude models of extraversion (between non-homebound and semi-homebound) as well as openness (between non-homebound and homebound groups), but not in adjusted models. It echoed the results of the ordinal logistics regression indicating associations between the two personality traits and homebound status.

Other than the above, we found no other statistically significant results in the rest of the combinations of homebound status group pairs (i.e., semi-homebound vs non-homebound, homebound vs non-homebound), personality traits, and models. It was understandable partly because individuals with homebound status changes only accounted for a small proportion of the sample – 195 participants (13%) became semi-homebound and 42 participants (3%) became homebound within 3 years. Although we tried to increase the number of incident cases by conducting cross-sectional analysis and prolonging the follow up time, we got similar results with primary analysis. Therefore, using the dataset with a large sample was still relatively limited considering our study aim. Nevertheless, the numbers of case proportion were noteworthy on population level, which may call for public health measures and policies [3]. In addition, homebound status was a relatively stable health status, so further longitudinal studies with a longer period of time (i.e., more than 6 years) may be able to clarify this association better.

One strength of this study is that we used a longitudinal dataset with a large sample (i.e., NHATS), which allows us to examine the predictive effects of personality traits on homebound status. Controlling of covariates and conducting of sensitivity analyses helps to provide relatively robust findings in this study. Some limitations should be noted in the current study. Since NHATS does not have pre-defined measures of homebound status, we needed to develop the measures. Those measures were limited to the questions of the pre-set items in the NHATS Mobility Questionnaire that are based on the reflections of the conditions of older adults’ activities in the last month. Recall and report biases may exist in all subjective items because participants may not recall the frequencies of their going outside accurately, and may be prone to report themselves to be more active and healthier than they actually were as per social desirability bias [47]. In addition, the 10-item personality measurement used in the NHATS is a simplified version of the original scale (60 items). This 10-item scale was used in large cohort studies but reliability and validity information were still limited. More studies using this scale may be needed to verify the results of our current study.

This study has important implications for research and clinical practice. It can provide useful information to guide plans for strategies aiming at screening, preventing, and inversing homebound status. For example, our findings indicated that a personality assessment can be an effective complementary tool to screen older adults and identify those adults who are at high risk of becoming homebound (e.g., people with low conscientiousness). This study also provided the scientific basis for establishing strategies to protect older adults from becoming homebound. Evidence showed that personality traits were associated with numerous health outcomes across the life span [48], including in old age [49]. Personality traits have been found to be amenable to clinical interventions [50]. Krasner et al. conducted an intensive educational program in mindful communication that has been shown to help to increase conscientiousness [51]. This mindfulness intervention focused on lowering one’s own reactivity to challenging experiences, cultivating one’s attention and awareness skills, and thus, helped reducing stress and improving one’s quality of life. These facts in combination with the results of our study can shed light on future research to prevent, decrease, and potentially inverse homebound status by targeted intervention programs in older adults with certain personality traits.

## Conclusions

Higher conscientiousness was associated with a lower likelihood of becoming homebound with and without adjusting for demographic and health-related covariates. Older adults with higher extraversion and higher openness were less likely to be homebound in crude models, but both associations became insignificant after adjusting for demographic and health-related covariates. Neuroticism was significantly associated with homebound status when adjusting for all demographic and health-related covariates. Further research is needed to confirm these relationships and find out possible mechanisms between these personality traits and homebound status. However, these findings can be a starting point to identify and intervene older adults with potentially risky personality traits of becoming homebound.

## Supplementary Information

**Additional file 1: Supplemental Table 1.** Correlation coefficient matrix of independent variables.

## Data Availability

The NHATS data analyzed in the current study are available for research purposes at www.nhats.org.

## Abbreviations

FFM: Five Factor Model
NHATS: National Health and Aging Trends Study
ADL: Activities of Daily Living
GAD-2: Generalized Anxiety Disorder-2
PHQ-2: Patient Health Questionnaire-2
ANOVA: Analysis of Variance
OR: Odd Ratio
CI: Confidence Interval
RRR: Relative Risk Ratio
SD: Standard Deviation
