# Supplementary material for: Longitudinal association between personality traits and homebound status in older adults: results from the National Health and Aging Trends Study
Source: BMC Geriatr. 2022 Feb 2;22:93. doi: 10.1186/s12877-022-02771-8 (PMC8812013; doi:10.1186/s12877-022-02771-8)
Supplement: Supplementary file 1 — Additional file 1: Supplemental Table 1. Correlation coefficient matrix of independent variables. [file 12877_2022_2771_MOESM1_ESM.docx]

Supplemental Table 1. Correlation coefficient matrix of independent variables.

| Variables | (1) | (2) | (3) | (4) | (5) | (6) | (7) | (8) | (9) | (10) | (11) | (12) | (13) | (14) | (15) | (16) | (17) |
| --- | --- | --- | --- | --- | --- | --- | --- | --- | --- | --- | --- | --- | --- | --- | --- | --- | --- |
| (1) Age | 1.000 |  |  |  |  |  |  |  |  |  |  |  |  |  |  |  |  |
| (2) Gender | -0.072  *** | 1.000 |  |  |  |  |  |  |  |  |  |  |  |  |  |  |  |
| (3) Race | -0.061  ** | -0.019 | 1.000 |  |  |  |  |  |  |  |  |  |  |  |  |  |  |
| (4) Education | -0.091  *** | 0.075  *** | -0.193  *** | 1.000 |  |  |  |  |  |  |  |  |  |  |  |  |  |
| (5) Living arrangement | -0.216  *** | 0.132  *** | 0.126  *** | -0.049  * | 1.000 |  |  |  |  |  |  |  |  |  |  |  |  |
| (6) Number of ADL difficulties | 0.076  *** | -0.014 | 0.019 | -0.086  *** | 0.003 | 1.000 |  |  |  |  |  |  |  |  |  |  |  |
| (7) Number of chronic illnesses | 0.039 | -0.130  *** | 0.052  ** | -0.094  *** | -0.020 | 0.093  *** | 1.000 |  |  |  |  |  |  |  |  |  |  |
| (8) Dementia | 0.096  *** | 0.015 | -0.002 | -0.083  *** | 0.031 | 0.096  *** | 0.076  *** | 1.000 |  |  |  |  |  |  |  |  |  |
| (9) Anxiety symptom | 0.019 | -0.081  *** | 0.010 | -0.086  *** | 0.023 | 0.094  *** | 0.079  *** | 0.066  ** | 1.000 |  |  |  |  |  |  |  |  |
| (10) Depressive symptom | 0.011 | 0.001 | 0.055  ** | -0.081  *** | 0.011 | 0.161  *** | 0.074  *** | 0.032 | 0.282  *** | 1.000 |  |  |  |  |  |  |  |
| (11) Pain | -0.033 | -0.107  *** | 0.056  ** | -0.032 | 0.011 | 0.131  *** | 0.218  *** | -0.012 | 0.074  *** | 0.052  ** | 1.000 |  |  |  |  |  |  |
| (12) Hospitalization | 0.049  * | 0.039 | -0.025 | -0.048  * | -0.022 | 0.110  *** | 0.174  *** | 0.052  ** | 0.042  * | 0.048  * | 0.100  *** | 1.000 |  |  |  |  |  |
| (13) Conscientiousness | -0.037 | -0.023 | -0.060  ** | 0.185  *** | -0.024 | -0.095  *** | -0.080  *** | -0.081  *** | -0.078  *** | -0.105  *** | -0.063  ** | -0.006 | 1.000 |  |  |  |  |
| (14) Extraversion | -0.062  ** | -0.093  *** | 0.022 | 0.036 | 0.005 | -0.022 | 0.029 | -0.043  * | -0.034 | -0.064  ** | 0.008 | 0.011 | 0.194  *** | 1.000 |  |  |  |
| (15) Neuroticism | -0.036 | -0.181  *** | -0.060  ** | -0.091  *** | 0.024 | 0.109  *** | 0.072  *** | 0.039 | 0.286  *** | 0.155  *** | 0.097  *** | 0.035 | -0.043  * | 0.005 | 1.000 |  |  |
| (16) Openness | -0.094  *** | 0.088  *** | 0.014 | 0.172  *** | 0.032 | 0.004 | -0.009 | -0.042  * | 0.009 | -0.027 | 0.033 | 0.000 | 0.274  *** | 0.330  *** | 0.002 | 1.000 |  |
| (17) Agreeableness | -0.023 | -0.185  *** | -0.024 | -0.020 | 0.044  * | -0.011 | 0.047  * | -0.056  ** | -0.004 | -0.084  *** | 0.027 | 0.017 | 0.222  *** | 0.392  *** | 0.059  ** | 0.240  *** | 1.000 |
| Note: **** p<0.01, ** p<0.05, * p<0.1* | | | | | | | | | | | | | | | | | |
